# Supplementary material for: Closed-loop error damping in human BCI using pre-error motor cortex activity
Source: bioRxiv. 2026 Feb 26:2026.02.25.707999. Preprint. [Version 1] doi: 10.64898/2026.02.25.707999 (PMC13160010; doi:10.64898/2026.02.25.707999)
Supplement: Supplement 2 [file NIHPP2026.02.25.707999v1-supplement-2.pdf]

bioRxiv preprint doi: https://doi.org/10.64898/2026.02.25.707999; this version posted February 26, 2026. The copyright holder for this preprint (which was not certified by peer review) is the author/funder, who has granted bioRxiv a license to display the preprint in perpetuity. It is made available under aCC-BY 4.0 International license.

5 Supplementary material

Video 1 : Trajectory during example trials from participant P2, without (blue, left) and with (orange, right) error modulation. Trajectories are accelerated 10 times.

bioRxiv preprint doi: https://doi.org/10.64898/2026.02.25.707999; this version posted February 26, 2026. The copyright holder for this preprint (which was not certified by peer review) is the author/funder, who has granted bioRxiv a license to display the preprint in perpetuity. It is made available under aCC-BY 4.0 International license.

Participant	Number of sets	Duration	Proportion of erroneous control
C2	16	245.68 ± 34.49s	50.11 ± 3.74 %
P2	17	260.23 ± 86.42s	37.93 ± 4.76 %
P3	4	197.0 ± 8.53s	36.48 ± 6.43 %
P4	7	158.95 ± 87.9s	30.46 ± 5.87 %

Supplementary Table 1: Experimental sets of 40 trials each used in sections 2.2 and 2.3. Durations and proportions of erroneous control are averaged over all sets for each participant.

bioRxiv preprint doi: https://doi.org/10.64898/2026.02.25.707999; this version posted February 26, 2026. The copyright holder for this preprint (which was not certified by peer review) is the author/funder, who has granted bioRxiv a license to display the preprint in perpetuity. It is made available under aCC-BY 4.0 International license.

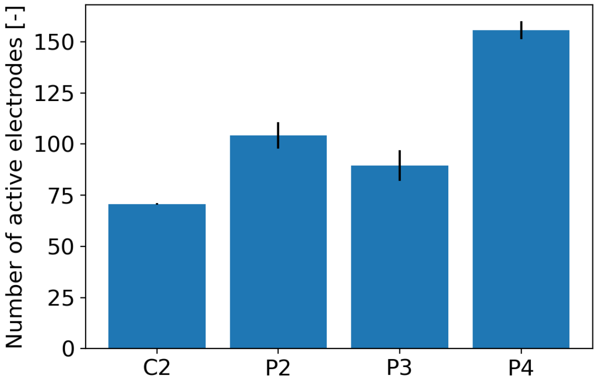

Supplementary Figure 1: Number of included electrodes (defined as the number of electrodes in both arrays for which the firing rate is above the set mean at least 5% of the set duration), averaged over all sets for each participant.

bioRxiv preprint doi: https://doi.org/10.64898/2026.02.25.707999; this version posted February 26, 2026. The copyright holder for this preprint (which was not certified by peer review) is the author/funder, who has granted bioRxiv a license to display the preprint in perpetuity. It is made available under aCC-BY 4.0 International license.

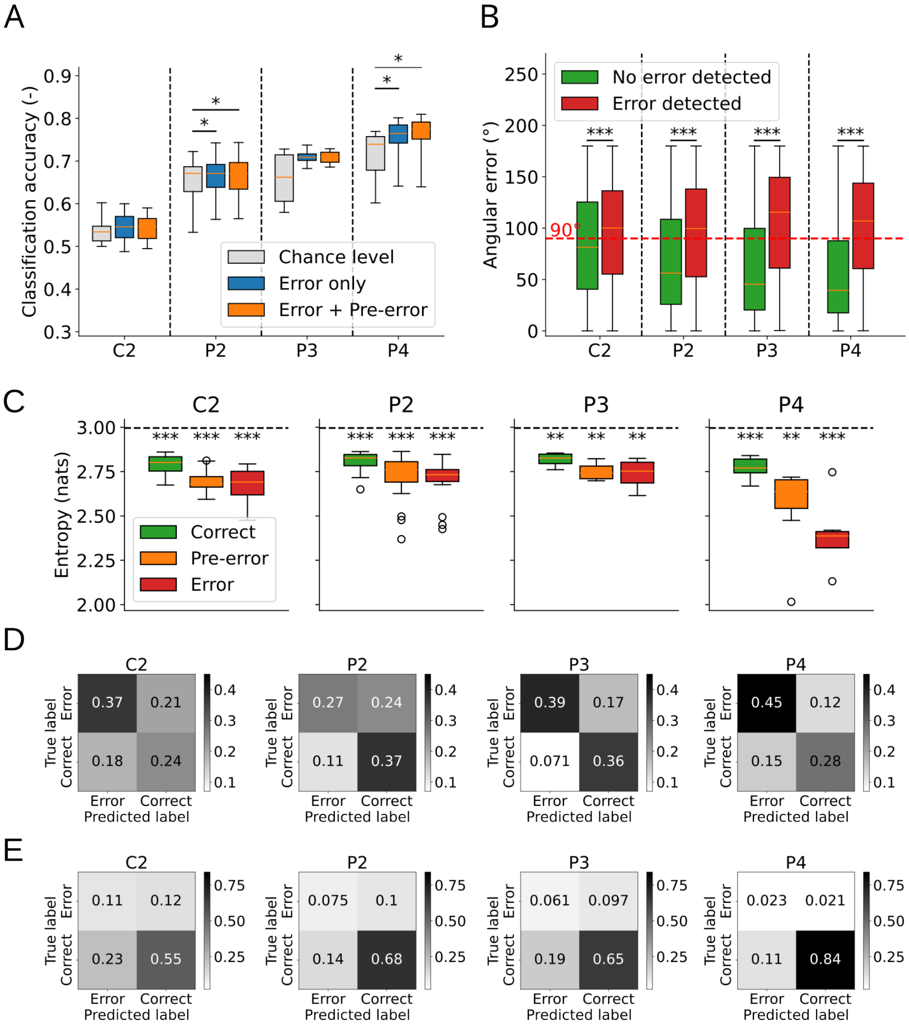

Supplementary Figure 2: A: Same results as in Fig. 2B but with cross-validation performed across targets (*Chance level* vs. *Error only*: C2: $p = 0.16$; P2: $p = 0.015$; P3: $p = 0.092$; P4: $p = 0.038$, one-sided paired t-test. *Chance level* vs. *Error + Pre-error*: C2: $p = 0.194$; P2: $p = 0.017$; P3: $p = 0.119$; P4: $p = 0.018$, one-sided paired t-test). B: Same results as in Fig. 2F but with cross-validation performed across targets (C2: $p < 0.001$; P2: $p < 0.001$; P3: $p < 0.001$; P4: $p < 0.001$, one-sided t-test). C: Entropies of the distribution of the cursor velocity direction in the correct, pre-error, and error windows, for all sets from each participant. The black dashed line represents the entropy of a uniform distribution, which is significantly higher than observed entropies (C2: $p < 0.001$, $p < 0.001$, $p < 0.001$; P2: $p < 0.001$, $p < 0.001$, $p < 0.001$; P3: $p = 0.002$, $p = 0.002$, $p = 0.006$; P4: $p < 0.001$, $p = 0.002$, $p < 0.001$, one-sample one-sided t-tests). D: Confusion matrices (averaged over all sets for each participant) for the direction bin containing the most erroneous epochs in each set (e.g. $225° - 270°$ for the set illustrated in Fig. 2G). E: Same results for the direction bin containing the least erroneous epochs in each set (e.g. $0° - 45°$ for the set illustrated in Fig. 2G).

bioRxiv preprint doi: https://doi.org/10.64898/2026.02.25.707999; this version posted February 26, 2026. The copyright holder for this preprint (which was not certified by peer review) is the author/funder, who has granted bioRxiv a license to display the preprint in perpetuity. It is made available under aCC-BY 4.0 International license.

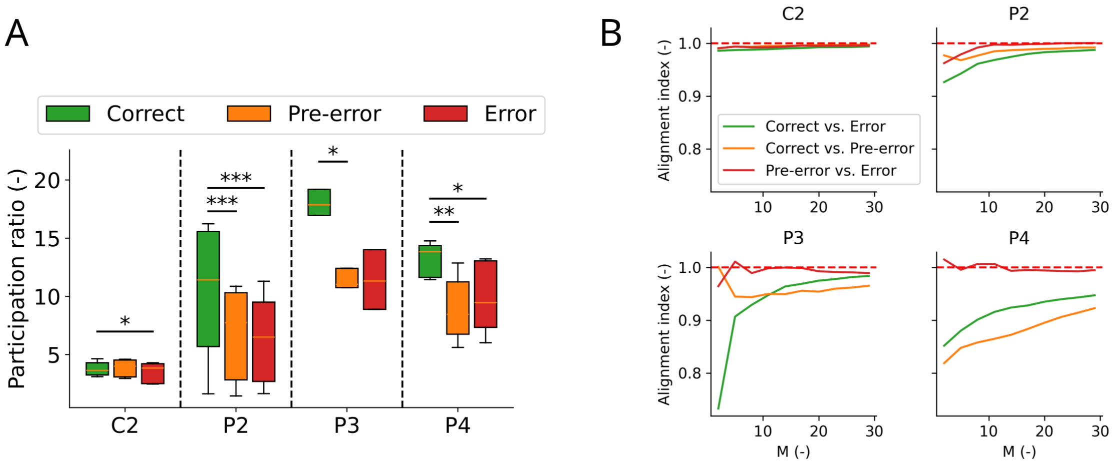

Supplementary Figure 3: A: Same results as in Fig. 3C but using the participation ratio (see Dimensionality computation) for estimating the dimensionality of each subspace. *Correct* vs. *Error*: C2: $p = 0.033$; P2: $p < 0.001$; P3: $p = 0.074$; P4: $p = 0.031$, one-sided paired t-test. *Correct* vs. *Pre-error*: C2: $p = 0.414$; P2: $p < 0.001$; P3: $p = 0.042$; P4: $p = 0.005$, one-sided paired t-test. B: Same results as in Fig. 3A but for different values of the parameter M (see Subspace alignment index).

bioRxiv preprint doi: https://doi.org/10.64898/2026.02.25.707999; this version posted February 26, 2026. The copyright holder for this preprint (which was not certified by peer review) is the author/funder, who has granted bioRxiv a license to display the preprint in perpetuity. It is made available under aCC-BY 4.0 International license.

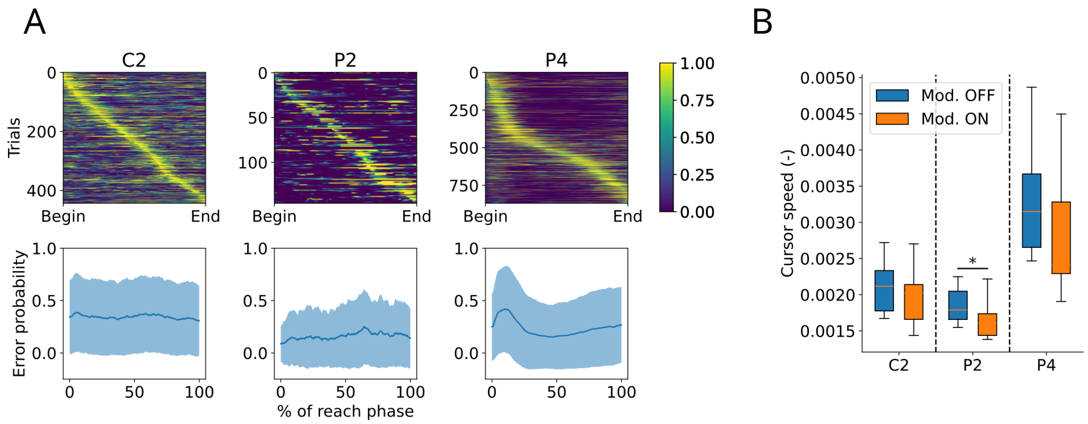

Supplementary Figure 4: A: Top row: Heatmap showing the error probability computed by the classifier for different trials (vertical axis) as a function of the trial completion (horizontal axis). Trials are ordered by peak error probability timepoint. Bottom row: same results, averaged over all trials. Shaded area: standard error. B: Cursor speed for all phases of the trials studied in section 2.4 for all three participants (C2: $p = 0.116$; P2: $p = 0.042$; P4: $p = 0.1$, one-sided t-test).

bioRxiv preprint doi: https://doi.org/10.64898/2026.02.25.707999; this version posted February 26, 2026. The copyright holder for this preprint (which was not certified by peer review) is the author/funder, who has granted bioRxiv a license to display the preprint in perpetuity. It is made available under aCC-BY 4.0 International license.

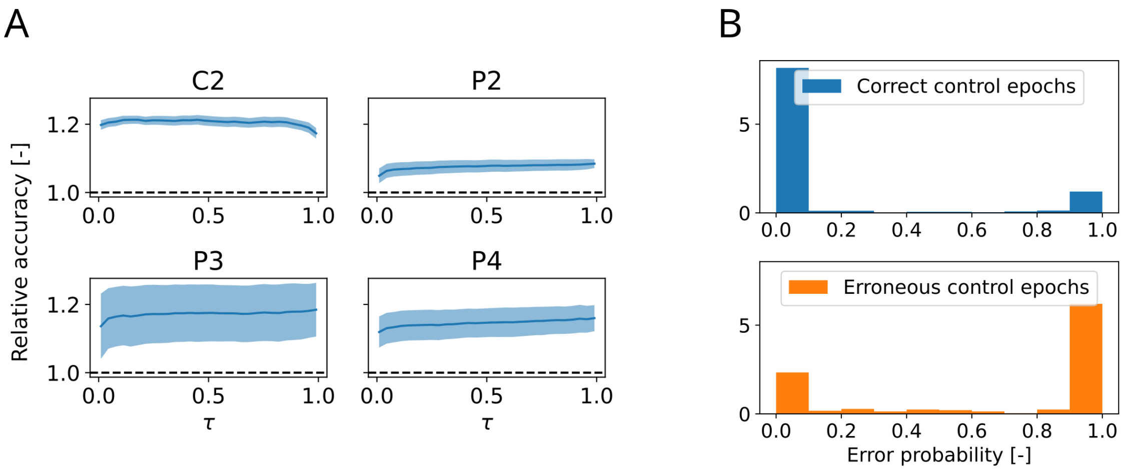

Supplementary Figure 5: A: Average classification accuracy (normalized by the chance level as defined in section 2.2) for all sessions of each participant as a function of the error detection threshold τ. Shaded area: standard error of the mean. B: Histograms of the error probability values computed by the classifier during epochs of correct (top) and erroneous control (bottom) for an example set from participant P4.
